# Lanreotide Autogel in the Treatment of Persistent Diarrhea following a Total Colectomy

**DOI:** 10.1155/2015/686120

**Published:** 2015-12-06

**Authors:** Patrick Schoeters, Karl De Pooter

**Affiliations:** ^1^Department of Gastroenterology, AZ Herentals, 2200 Herentals, Belgium; ^2^Department of Gastrointestinal Surgery, AZ Herentals, 2200 Herentals, Belgium

## Abstract

Diarrhea is one of the most common complications following colectomy in patients with slow transit constipation (STC). Early postoperative diarrhea is usually treated with opioid agonists; however, to date, published data on the management of persistent diarrhea after colectomy for STC are scarce. Here, we report a case of severe diarrhea after a total colectomy with ileorectal anastomosis. One year after the surgery, the patient presented with persistent diarrhea. Treatment with a long-acting somatostatin analogue, lanreotide Autogel, was initiated. One month after the first injection of lanreotide Autogel the diarrhea was resolved. The patient's stool transit was markedly improved (type 4 or type 5 according to the Bristol Stool Chart compared to type 7 before the treatment), positively affecting the patient's quality of life (mean score of 2.1 on the Irritable Bowel Syndrome Quality of Life questionnaire compared to 3.9 before the treatment). This case report describes a successful use of lanreotide Autogel in a patient with persistent diarrhea after a total colectomy.

## 1. Introduction

Chronic constipation is one of the most common gastrointestinal conditions characterized by infrequent bowel movements, presence of hard stools, an excessive time to evacuate, straining, and sensation of incomplete bowel evacuation [1]. Recent Rome III Diagnostic Criteria for Functional Constipation defined constipation as <3 bowel movements per week and the occurrence of specific bowel symptoms, that is, straining, lumpy/hard stools, incomplete evacuation, sensation of anorectal obstruction, and manual maneuvers for defecation in >25% of defecations [2–4]. Slow transit constipation (STC) is a functional colonic disorder characterized by reduced frequency of high-amplitude propagated contractions and severe impairment of colonic motor activity that, in some cases, may lead to a true colonic inertia [5–8]. The reported rates of prevalence of chronic constipation vary by geographic region and disease definition from 1% to almost 40%; a recent meta-analysis based on 41 studies reported a pooled prevalence of 14% [7, 9–14]. Patients with STC represent 15–30% of all constipated patients; STC is most common in young women [8, 15]. Typical treatment options for patients with STC include dietary changes, enemas, laxatives, and pharmacologic therapy; other options such as biofeedback, sacral nerve stimulation, and surgery may be considered in patients with STC who are resistant to medical therapy [6, 8, 15–18]. Total colectomy with ileorectal anastomosis is the most widely used surgical procedure for patients with STC who do not respond to conventional treatments [15, 17, 19–22]. Diarrhea is one of the most common complications following colectomy and has been reported in up to 46% of patients who underwent colectomy [17, 19–21, 23–28]. During the adaptation phase following surgery, administration of fiber, motility agents (loperamide, diphenoxylate and atropine sulfate, or codeine), and binders (cholestyramine) may help in reducing bowel frequency [17, 23]. Although postoperative diarrhea is usually transient and can be controlled with medication, in some patients, failure of intestinal adaptation may lead to intractable diarrhea [17]. Here, we report the successful use of a long-acting somatostatin analogue, lanreotide, in the treatment of persistent diarrhea after a total colectomy.

## 2. Case Report

In July 2013, a 22-year-old woman presented with serious constipation. Between 2003 and 2005, she had been suffering from constipation and urine retention. In 2011, the patient had a car accident, after which she developed a whiplash and migraine, which was treated with valproate and carbamazepine. In 2012, she was diagnosed with reflux esophagitis grade A, treated with pantoprazole (40 mg/day), resolor (2 mg/day), otilonium bromide (40 mg 3x/day), mesalazine (1 g 3x/day), questran (4 g 3x/day), and loperamide (2 mg 3x/day). Upon admission to the hospital in July 2013, the patient was diagnosed with STC, confirmed by a radiopaque marker ingestion study which demonstrated a marked delay in the colonic transit time and by functional pelvic examination showing reasonably good sphincter pressures but incomplete relaxation of the pelvic floor. The patient was treated with prucalopride and pelvic floor training. However, the patient's symptoms progressed despite the medical treatment. Therefore, a decision of laparoscopic total colectomy with ileorectal anastomosis completed with biofeedback training of the pelvic floor was made. The patient underwent surgery under general anesthesia in July 2013. The procedure was successful and no complications during the surgery were recorded. Following the surgery, a smooth recovery with slow mobilization of the gastrointestinal transit was noted. The patient left the hospital in a good general condition with dietary advice 10 days following the surgery.

Six months after the surgery, the patient developed diarrhea (6–8 episodes/day), which was treated with otilonium bromide, cholestyramine, mesalazine, and loperamide. However, the treatment was unsuccessful. In May 2014, the patient presented to our hospital with continuous refractory diarrhea defined as >3 stools/day and nonresponsiveness to standard antidiarrheal therapy. Upon admission, the patient completed the Irritable Bowel Syndrome Quality of Life (IBS-QoL) questionnaire consisting of 34 questions describing the bowel problems experienced during the past 30 days [29, 30]. According to a 1–5 scale, where 1 indicates “not at all,” 2 indicates “slightly,” 3 indicates “moderately,” 4 indicates “quite a bit,” and 5 indicates “extremely/a great deal,” the patient assigned a score of 4 or 5 to the majority of her bowel problems; the mean score at admission was 3.9 (Table 1). The patient's stool was classified as type 7 according to the Bristol Stool Chart [7]. Infectious or inflammatory nature of the diarrhea was excluded by gastroscopy and ileoscopy as well as negative coprocultures. The diarrhea was generally accompanied by mild bowel spasms, but no mucus or blood was seen in the stools. Endoscopy at ileorectal anastomosis revealed small erosion at the anastomosis. Treatment with a short-acting somatostatin analogue, octreotide, administered subcutaneously for 3 days at 0.1 mg/day was initiated. Following 3 octreotide administrations, a mild response to treatment was observed; however, due to practical considerations, the treatment was switched to the long-acting somatostatin analogue, lanreotide, administered subcutaneously every 28 days. Lanreotide Autogel was administered as monthly injections at 90 mg dose during 5 months. Following the 1st injection the patient's diarrhea had improved; however, after the 5th injection, the diarrhea had worsened again. Therefore, the lanreotide dose was increased to 120 mg. Following the 1st injection of lanreotide Autogel 120 mg, the diarrhea was resolved (2 stools/day) and the patient's stool was classified as type 4 or type 5 stool on the Bristol Stool Chart. Furthermore, the patient's QoL had also improved, as indicated by lower scores she assigned to her bowel problems in the IBS-QoL questionnaire after the treatment with lanreotide Autogel 120 mg (mean score 2.1, Table 1). Lanreotide Autogel 120 mg was administered during 5 months; the patient follow-up at 5 months after the 1st lanreotide Autogel 120 mg administration revealed no diarrhea recurrence. At the end of this report, the patient's treatment with lanreotide Autogel 120 mg was still ongoing.

## 3. Discussion

Chronic constipation is a frequently encountered disorder in clinical practice. Most constipated patients benefit from standard medical approaches. However, these therapies fail in some patients. Colectomy with an ileorectal anastomosis aiming at increasing bowel movement frequency is the most widely accepted operative technique for patients with STC refractory to conventional treatments. However, diarrhea is one of the most common postoperative complications after colectomy for STC affecting up to 46% of patients [17, 19–21, 23–28]. Diarrhea after colectomy might severely affect a patient's QoL, although it generally resolves after one year following the surgery [15, 24]. Treatment of early postoperative diarrhea with diphenoxylate/atropine and loperamide has been reported [28]. However, the published reports on the management of persistent postcolectomy diarrhea are scarce.

Here, we describe the successful use of a long-acting somatostatin analogue, lanreotide, in the management of persistent diarrhea following a total colectomy. Following the administration of lanreotide Autogel, the patient's diarrhea significantly improved and was ultimately resolved within 28 days after treatment initiation. Following the treatment, the patient's stool form was classified as type 4 or type 5 according to the Bristol Stool Chart, compared to type 7 before the lanreotide Autogel 120 mg administration, indicating that this treatment was effective. The use of somatostatin analogues, a short-acting octreotide and a long-acting lanreotide, in the control of diarrhea has been also previously reported in patients with carcinoid tumors [31–34], diabetes [35], and idiopathic refractory diarrhea [36]. Somatostatin plays a regulatory function in several organs and systems, including the gastrointestinal tract, where it inhibits the gastrointestinal motility and colonic fluid secretion [37]. Although the mechanism by which somatostatin analogues affect bowel function has not yet been fully elucidated, it is likely that somatostatin analogues act by inhibiting the intestine hormone secretion, lengthening the intestinal transit time, and increasing the water absorption [38]. In this report, the improved transit following the lanreotide Autogel administration positively impacted the patient's QoL. In the absence of a specific questionnaire that could be used to assess the QoL of patients with persistent diarrhea, we used the IBS-QoL questionnaire that has been widely used in IBS patients [29, 30]. This questionnaire has been also used in a recent multicenter, prospective study evaluating the effect of lanreotide Autogel 120 mg in 36 patients with idiopathic refractory diarrhea, which showed improved symptoms of diarrhea and QoL upon the treatment (Medard; ClinicalTrials.gov NCT00891371) [36]. These and our results suggest that lanreotide Autogel is effective in the management of persistent refractory diarrhea.

## Figures and Tables

**Table 1:** Irritable Bowel Syndrome Quality of Life questionnaire score before and after the lanreotide Autogel treatment.

IBS-QoL question	Before lanreotide	After lanreotide
(Q1) I feel helpless because of my bowel problems.	5	2
(Q2) I am embarrassed by the smell caused by my bowel problems.	4	2
(Q3) I am bothered by how much time I spend on the toilet.	5	2
(Q4) I feel vulnerable to other illnesses because of my bowel problems.	3	2
(Q5) I feel fat/bloated because of my bowel problems.	5	2
(Q6) I feel like I'm losing control of my life because of my bowel problems.	5	2
(Q7) I feel my life is less enjoyable because of my bowel problems.	5	2
(Q8) I feel uncomfortable when I talk about my bowel problems.	5	3
(Q9) I feel depressed about my bowel problems.	4	2
(Q10) I feel isolated from others because of my bowel problems.	5	2
(Q11) I have to watch the amount of food I eat because of my bowel problems.	4	3
(Q12) Because of my bowel problems, sexual activity is difficult for me.	4	3
(Q13) I feel angry that I have bowel problems.	4	2
(Q14) I feel like I irritate others because of my bowel problems.	5	2
(Q15) I worry that my bowel problems will get worse.	5	2
(Q16) I feel irritable because of my bowel problems.	4	3
(Q17) I worry that people think I exaggerate my bowel problems.	3	2
(Q18) I feel I get less done because of my bowel problems.	4	2
(Q19) I have to avoid stressful situations because of my bowel problems.	4	2
(Q20) My bowel problems reduce my sexual desire.	3	2
(Q21) My bowel problems limit what I can wear.	3	1
(Q22) I have to avoid strenuous activity because of my bowel problems.	3	2
(Q23) I have to watch the kind of food I eat because of my bowel problems.	3	3
(Q24) Because of my bowel problems I have difficulty being around people I do not know well.	4	2
(Q25) I feel sluggish because of my bowel problems.	4	2
(Q26) I feel unclean because of my bowel problems.	4	3
(Q27) Long trips are difficult for me because of my bowel problems.	4	3
(Q28) I feel frustrated that I cannot eat when I want because of my bowel problems.	2	2
(Q29) It is important to be near a toilet because of my bowel problems.	5	3
(Q30) My life revolves around my bowel problems.	4	2
(Q31) I worry about losing control of my bowels.	4	2
(Q32) I fear I won't be able to have a bowel movement.	1	1
(Q33) My bowel problems are affecting my closest relationships.	2	1
(Q34) I feel that no one understands my bowel problems.	2	2

Mean score	**3.9**	**2.1**

IBS-QoL, Irritable Bowel Syndrome Quality of Life.

Before lanreotide, score before lanreotide Autogel treatment.

After lanreotide, score after lanreotide Autogel treatment.

Q, question.
